# Evaluation of Blended Poly(3-hydroxybutyrate-*co*-3-hydroxyhexanoate) Properties Containing Various 3HHx Monomers

**DOI:** 10.3390/polym16213077

**Published:** 2024-10-31

**Authors:** Nara Shin, Su Hyun Kim, Jinok Oh, Suwon Kim, Yeda Lee, Yuni Shin, Suhye Choi, Shashi Kant Bhatia, Jong-Min Jeon, Jeong-Jun Yoon, Jeong Chan Joo, Yung-Hun Yang

**Affiliations:** 1 Department of Biological Engineering, College of Engineering, Konkuk University, Seoul 05029, Republic of Korea; dksk71@naver.com (N.S.); gsm06136@naver.com (S.H.K.); xmfvm@naver.com (J.O.); rlatn990@naver.com (S.K.); karecurry@konkuk.ac.kr (Y.L.); sdbsdl0526@naver.com (Y.S.); suhye0823@konkuk.ac.kr (S.C.); shashikonkukuni@konkuk.ac.kr (S.K.B.); 2 Institute for Ubiquitous Information Technology and Application, Konkuk University, Seoul 05029, Republic of Korea; 3 Green & Sustainable Materials R&D Department, Research Institute of Clean Manufacturing System, Korea Institute of Industrial Technology (KITECH), Cheonan-si 31056, Republic of Korea; j2pco@kitech.re.kr (J.-M.J.); jjyoon@kitech.re.kr (J.-J.Y.); 4 Department of Chemical Engineering, Kyung Hee University, Yongin-si 17104, Republic of Korea

**Keywords:** bioplastics, polyhydroxyalkanoate, poly(3-hydroxybutyrate-*co*-3-hydroxyhexanoate), polymer blends

## Abstract

Polyhydroxyalkanoate (PHA), specifically poly(3-hydroxybutyrate-*co*-3-hydroxyhexanoate) (P(3HB-*co*-3HHx), PHBHHx) with physical properties governed by the 3-hydroxyhexanoate (3HHx) mole fraction, is a promising bioplastic. Although engineered strains used to produce P(3HB-*co*-3HHx) with various 3HHx mole contents and fermentation techniques have been studied, mass production with specific 3HHx fractions and monomers depends on the batch, supply of substrates, and strains, resulting in the time-consuming development of strains and complex culture conditions for P(3HB-*co*-3HHx). To overcome these limitations, we blended poly(3-hydroxybutyrate) [(P(3HB), produced from *C. necator* H16] and P(3HB-*co*-20 mol%3HHx) [from *C. necator* 2668/pCB81] to prepare films with various 3HHx contents. We evaluated the molecular weight and physical, thermal, and mechanical properties of these films and confirmed the influence of the 3HHx monomer content on the mechanical and thermal properties as well as degradability of the blended P(3HB-*co*-3HHx) films containing various 3HHx mole fractions, similar to that of original microbial-based P(3HB-*co*-3HHx). Moreover, the degradation rate analyzed via *Microbulbifer* sp. was >76% at all blending ratios within 2 days, whereas a weaker effect of the 3HHx mole fraction of the blended polymer on degradation was observed. P(3HB-*co*-3HHx) could be produced via simple blending using abundantly produced P(3HB) and P(3HB-*co*-20 mol%HHx), and the resulting copolymer is applicable as a biodegradable plastic.

## 1. Introduction

The use of biodegradable bioplastics has garnered considerable interest in addressing the growing concerns regarding environmental pollution caused by conventional plastic materials [1]. These eco-friendly alternatives present promising solutions for mitigating the harmful impacts of accumulating plastic waste on the ecosystem and human health [2]. The use of biodegradable bioplastics is a crucial strategy for promoting sustainability and conservation of natural resources [3].

Among bioplastics, polyhydroxyalkanoates (PHAs) are produced from renewable sources such as microorganisms, are ecofriendly, and have properties similar to those of conventional plastics [4]. PHA can be produced by various microorganisms including bacteria (such as *Cupriavidus necator*, *Bacillus megaterium*, *Alcaligenes latus*, and *Pseudomonas putida*), fungi from the genera *Aspergillus*, and prokaryotes such as *Methanotrophs* and *Cyanobacteria* [5,6,7,8,9,10]. Poly(3-hydroxybutyrate) (P(3HB)), which is naturally produced by *Cupriavidus necator*, is a PHA with a short chain length, indicating that its polymer units consist of relatively few carbon atoms (typically 1–3) [11]. The daily use of P(3HB) is increasing, especially in products such as packaging materials, disposable items, and medical applications including sutures and drug delivery systems, making it a more sustainable alternative to conventional plastics [12,13,14]. However, the application of P(3HB) is limited by inherent drawbacks such as brittleness, which constrains its mechanical resilience and impacts resistance [15,16]. Moreover, its susceptibility to degradation at high temperatures hinders its application in systems requiring thermal stability [17]. Conversely, poly(3-hydroxybutyrate-*co*-3-hydroxyhexanoate) (P(3HB-*co*-3HHx)) is a copolymer composed of 3-hydroxybutyrate (3HB) and 3-hydroxyhexanoate (3HHx), which is commonly found in recombinant *Cupriavidus necator* [18]. The relative of the 3HB:3HHx molar ratio significantly influences P(3HB) characteristics [19]. Functioning as a plasticizer, P(3HB-*co*-3HHx) improves the physical characteristics of P(3HB) through various means. P(3HB-*co*-3HHx) decreases the crystallinity of P(3HB), resulting in increased flexibility and durability [20]. Additionally, this copolymer enhances the processability of P(3HB) by reducing its viscosity, thereby facilitating simpler molding and shaping during the manufacturing processes [21]. By varying the mole fraction of monomers, properties similar to those of polyethylene (PE) and polypropylene (PP) can be achieved [22]. P(3HB-*co*-3HHx) is biodegradable and possesses robust thermal and mechanical properties, making it a potential material with a wide range of applications and an ecofriendly alternative to plastic [23,24]. Therefore, efficient production methods for P(3HB-*co*-3HHx) are required; the current approaches include using various bacterial strains, such as *E. coli*, *C. necator* sp., *Aeromonas* sp., *Halomonas* sp., and *Pseudomonas* sp. (Table 1).

**Figure 1:**
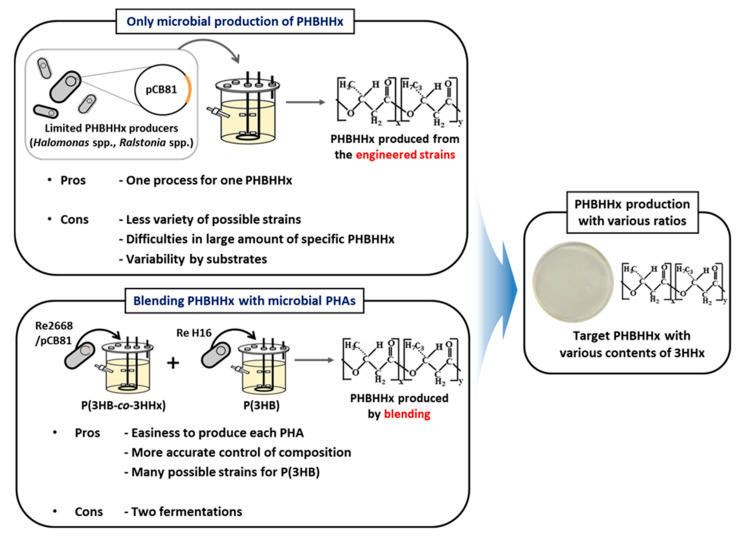
Schematic of the methods for producing P(3HB-*co*-3HHx) with various 3-hydroxyhexanoate (3HHx) mole fractions.

The methods used for controlling the mole fractions of 3HHx to produce P(3HB-*co*-3HHx) with desired characteristics include culturing under various conditions using bacteria or blending P(3HB) and P(3HB-*co*-3HHx) at specific ratios (Figure 1). To produce P(3HB-*co*-3HHx) using bacteria, engineered bacterial strains capable of synthesizing both 3HB and 3HHx, such as *Halomonas* spp. and *Ralstonia* spp., are cultured in a fermentation a fermentation medium [31]. By controlling the substrates, the relative 3HB:3HHx ratios can be controlled, enabling the production of copolymers with various mole fractions of 3HHx [32]. The method based on strain engineering has the advantage of producing P(3HB-*co*-3HHx) with different 3HHx mole fractions from a specific substrate via one process [33]. However, the strains capable of producing different P(3HB-*co*-3HHx) copolymers are limited. Moreover, it is difficult to produce abundant P(3HB-*co*-3HHx) with a constant 3HHx mole fraction via the fermentation steps owing to the large number of substrates [34]. In the blending method, microorganisms are used to produce P(3HB) and P(3HB-*co*-3HHx) separately; the polymers are then blended to produce P(3HB-*co*-3HHx) with various 3HHx mole fractions. This method requires two fermentation steps to produce each of the two types of polymers; however, there are many strains that can produce P(3HB) required for blending, such as *C. necator* sp., *Pseudomonas* sp., and *Bacillus* sp. [35,36,37,38,39]. In addition, the accurate composition of the blend can be controlled, and P(3HB-*co*-3HHx) production is relatively easy because it does not involve substrate changes.

Moreover, the application of mixed microbial cultures to produce P(3HB-*co*-3HHx) reportedly demonstrated cost reduction and resource utilization [40]; however, it is difficult to control the composition and maintain the purity of the product using this approach [41]. Furthermore, in mixed microbial cultures, because multiple microorganisms present in the sludge simultaneously interact to produce polymers, the metabolic pathways and reaction rates of each microorganism may differ. Thus, accurate results cannot be obtained, thereby resulting in limited reproducibility of the production process [42]. To overcome these limitations, the strategy of separately producing PHB and P(3HB-*co*-3HHx) and then blending them was suggested, because blending can be better controlled, enhancing the reproducibility of the production process [43]. This type of approach was previously difficult to implement because obtaining a strain that produces P(3HB-*co*-3HHx) containing a high mole fraction of 3HHx was difficult. Nevertheless, as a high mole fraction of 3HHx can be achieved in abundance using strains such as *C. necator* H16 and Re2668/pCB81 [44], this modified blending strategy is reasonable within the 3HHx mole fraction range of the strain.

Therefore, in this work, P(3HB) and P(3HB-*co*-3HHx) were blended to compose P(3HB-*co*-3HHx) containing various mole fractions of 3HHx. The changes in the molecular weight and mechanical and thermal properties with the increase in the 3HHx mole fraction were investigated. In addition, *Microbulbifer* sp., which is a PHA-degrading strain known to degrade P(3HB) by approximately 40% in 2 days and approximately 100% in 7 days, was used to perform degradation tests and confirm the biodegradability of P(3HB-*co*-3HHx) with various mole fractions of 3HHx produced by blending [45]. We compared the properties of blended P(3HB-*co*-3HHx) and microbial-derived original P(3HB-*co*-3HHx), and evaluated the physical properties and thermal properties of blended P(3HB-*co*-3HHx). In addition, we propose a blending method using P(3HB-*co*-3HHx) to be useful across industries, similar to P(3HB-*co*-3HHx) produced solely by microorganisms.

## 2. Materials and Methods

### 2.1. Bacterial Strains

The bacterial strains and plasmids used in this study are listed in Table 2. Here, *C. necator* H16 and Re2668/pCB81 were precultured in 5 mL of tryptic soy broth (TSB) (Seoul, Republic of Korea) supplemented with appropriate antibiotics (10 μg/mL gentamicin and 50 μg/mL kanamycin) for 24 h at 30 °C. Cell growth was monitored by measuring the optical density (OD) at 595 nm, starting from an initial ${OD}_{595}$ of 0.05. Furthermore, P(3HB) was produced using wild-type *C. necator* H16, and P(3HB-*co*-3HHx) was produced using the engineered *C. necator* 2668/pCB81. *Microbulbifer* sp. SOL66 was used to degrade P(3HB-*co*-3HHx). These strains were stored in 50% glycerol at −80 °C.

### 2.2. Fed-Batch Fermentation to Produce P(3HB) and P(3HB-co-3HHx)

Precultures for the fermenter were prepared at a volume of 50 mL (TSB) in two 250 mL baffled flasks at 30 °C for 24 h. Each culture was centrifuged at 3511× *g* at 4 °C, and the cell pellets were washed twice with 10 mL of distilled water (DW). Fed-batch fermentation was performed using a 3 L working volume in a 5 L fermenter (CNS, Seoul, Republic of Korea). Fermentation was conducted using the *Ralstonia eutropha* minimal medium (ReMM). The ReMM initially had a pH of 6.8 and contained 0.45 g/L $K_{2}{SO}_{4}$, 4.0 g/L ${NaH}_{2}{PO}_{4}$, 0.39 g/L ${MgSO}_{4}$, 4.6 g/L ${Na}_{2}{PO}_{4}$, 62 mg/L ${CaCl}_{2}$, and 1 mL/L of a trace element solution that included 15 g/L ${FeSO}_{4}\cdot {7H}_{2}O$, 2.4 g/L ${MnSO}_{4}\cdot H_{2}O$, 2.4 g/L ${ZnSO}_{4}\cdot {7H}_{2}O$, and 0.48 g/L ${CuSO}_{4}\cdot {5H}_{2}O$ dissolved in 0.1 M hydrochloric acid. For the production of P(3HB) and P(3HB-*co*-3HHx), 2% (*w*/*v*) fructose was commonly used as the carbon source, 1 g/L of urea was used as the nitrogen source, and 5% soybean oil (*w*/*v*) was added for the production of P(3HB-*co*-3HHx). The pH (6.75–6.85) of solution was adjusted using NaOH and HCl. For the main culture, 10% seed-culture (*v*/*v*) was inoculated. Re2668/pCB81 and *C. necator* H16 cells were harvested at 132 h and 140 h, respectively. Additionally, 5% of soybean oil was gradually supplied to 10–20 h, which is the exponential phase. The pH of 6.75–6.85 of the solution was adjusted using NaOH and HCl. The dissolved oxygen (DO) level was set at 20%. The initial stirring rate was 200 rpm and was increased to 600 rpm to maintain the DO level during culture [48]. Unless otherwise specified, the medium components were purchased from Sigma-Aldrich (St. Louis, MO, USA).

### 2.3. Preparation of P(3HB) Blending with P(3HB-co-20 mol%3HHx)

To recover P(3HB) and P(3HB-*co*-3HHx) from fermenter cultivation, the complete microbial culture was harvested after 140 h, centrifuged at 4 °C and 3500 rpm for 20 min, and then rinsed with distilled water. The resulting biomass was transferred to a glass vial and dried for 32 h by lyophilization to determine dry cell weight (DCW). Each lyophilized cell was dissolved in 100 mL high-performance liquid chromatography (HPLC)-grade chloroform and lysed for 1 h at 60 °C in a water bath [49].

When each polymer was dissolved and the P(3HB) and P(3HB-*co*-3HHx) polymers in chloroform were extracted, 50 mL of the extract was dispensed into the plates, and chloroform was completely evaporated in a fume hood to form the films required for blending. After determining the amount of each film for blending by considering the molecular number and weight of the monomers in each polymer, the films were dissolved in chloroform and evaporated in a fume hood to obtain the blended film. Because a large amount of P(3HB) and P(3HB-*co*-3HHx) were extracted, the step of adding the concentrated chloroform solution to cold methanol was omitted for convenience of extraction. The 3HHx content of each film was determined via gas chromatography (GC) [50].

### 2.4. Gel Permeation Chromatography (GPC)

The molecular weights of the original P(3HB) and P(3HB-*co*-3HHx) from microorganisms and blended P(3HB-*co*-3HHx) samples were determined via gel permeation chromatography (GPC) (YoungIn ChroMass, Anyang, Republic of Korea). The samples were dissolved in 2 mL of chloroform and heated at 60 °C for 1 h. The resulting solution was filtered using a syringe polyvinylidene fluoride (PVDF) filter (0.2 μm pore size; Chromdisc, Daegu, Republic of Korea). The analysis was conducted using an HPLC apparatus, including a loop injector (Rheodyne 7725i), an isocratic pump with dual heads (YL9112), a column oven (YL9131), columns (Shodex, K-805, 8.0 mm I.D. × 300 mm; Shodex, K-804, 8.0 mm I.D. × 300 mm), and a refractive index detector (YL9170) [51]. A 60 μL sample was injected for analysis, and chloroform served as the mobile phase with a flow rate of 1.0 mL/min and 35 °C. The data were processed using Clarity software version 8.3 specifically designed for a single YL HPLC instrument (YoungIn ChroMass). The weight average molecular weight ($M_{w}$), number average molecular weight ($M_{n}$), and dispersity (Đ) were determined relative to polystyrene standards ranging from 5000 to 2,000,000 g/mol.

### 2.5. Differential Scanning Calorimetry (DSC)

The thermal properties of the PHA polymers were measured using a NEXTA DSC 200 (Hitachi, Tokyo, Japan). The experiment was conducted in an $N_{2}$ atmosphere at temperatures ranging from −60 to 180 °C, with a heating and cooling rate of 10 °C/min [52]. The polymer film samples were cut into small pieces and placed in aluminum pans. Then, inert nitrogen and approximately 7 mg samples were heated in the pans in an inert nitrogen atmosphere and cooled at a rate of −263.1 °C ${min}^{-1}$ from −60 to 180 °C [53]. The melting temperature ($T_{m}$), glass transition temperature ($T_{g}$), enthalpy change (Δ*H*), and cold crystallization temperature ($T_{c}$) were determined on the basis of the second heating.

### 2.6. Analysis of Mechanical Properties

The mechanical properties of the original P(3HB) and P(3HB-*co*-3HHx) from microorganisms and blended P(3HB-*co*-3HHx) samples were tested using a universal testing machine (UTM) EZ-SX (Shimadzu, Kyoto, Japan). The test specimens were cut into a rectangular shape (20 mm wide × 60 mm long) and conditioned according to the ASTM D882 standard [54,55,56,57]. The tests were conducted at a crosshead speed of 10 mm/min [58]. The elongation at break (EL) was calculated using Equation (1):

(1) $$EL=\frac{d_{after}-d_{before}}{d_{before}}\times 100$$

where *d* is the distance between the grips holding the sample before and after breaking.

### 2.7. Spectroscopy

#### 2.7.1. Fourier Transform Infrared Spectroscopy (FT-IR)

The functional groups of original P(3HB-*co*-3HHx) and blended P(3HB-*co*-3HHx) were detected by Fourier transform infrared (FT-IR) spectroscopy. The FT-IR analysis was performed using an FT-IR spectrometer (Thermo Scientific Nicolet IR200, Waltham, MA, USA). The spectra were achieved in the range of 4000 to 500 ${cm}^{-1}$ with a resolution of 4 cm^−1^ [59].

#### 2.7.2. H Nuclear Magnetic Resonance (^1^H NMR) Spectroscopy

The original and blended P(3HB-*co*-3HHx) were analyzed by ^1^H NMR spectroscopy. Samples were dissolved in deuterated chloroform (CDCl_3_) at 8 mg/mL to apply for NMR analysis. NMR spectra were recorded on a Bruker Avance 600 spectrometer (Bruker Co., Billerica, MA, USA). ^1^H spectra were obtained at 500 MHz at room temperature [60]. Chemical shifts such as resonance signals (δ) were obtained in ppm compared to the outstanding signals of CDCl_3_ as the internal reference (^1^H NMR: 7.26 ppm). At the same time, adamantane was used as an external standard.

#### 2.7.3. UV-Visible Spectroscopy (UV-Vis)

The PHA content was measured using the UV-Vis [61]. A spectrum was recorded between 200 and 800 nm with a 10 cm quartz cuvette [62]. The lamp of the device provides monochromatic light in the visual and UV range.

### 2.8. Clear Zone Test for Selecting P(3HB-co-3HHx) Degrading Strain

The clear zone test was conducted to confirm the degradation of the blended P(3HB-*co*-3HHx) by microorganisms. PHB-degrading *Microbulbifer* sp. SOL66 was used to confirm the degradability of P(3HB-*co*-3HHx). To prepare a solid plate containing P(3HB-*co*-3HHx) at various 3HHx mole fractions, 1 g of each film was added to 40 mL of dichloromethane (DCM) and dissolved in a water bath at 60 °C until the films were completely dissolved [63]. The lysate was diluted with 100 mL of DW, which was followed by the addition of 2 mL of 2% Sarkosyl NL at the interface. The mixture was sonicated using a Vibra-Cell VCX500 (Sonics & Materials, Inc., Nemolown, CT, USA) with 15 s of pulsing at 30% amplitude for 10 min. Subsequently, 2% agarose and 1 g/L bioplastic emulsion were added to the marine broth (MB; Difco Laboratories, Detroit, MI, USA) medium, and the mixture was sterilized by autoclaving at 121 °C for 15 min [64]. The strains were cultured in an MB liquid medium for 24 h at 37 °C. The 6 mm paper discs (Toyo Roshi Kaisha, Tokyo, Japan) were then placed on a plate containing P(3HB-*co*-3HHx), and 10 μL of the cultured cells were inoculated onto the paper discs and incubated at 37 °C for 2 days.

### 2.9. Gas Chromatography-Mass Spectrometry (GC-MS) Analysis

The amount of P(3HB-*co*-3HHx) with various mole fractions of 3HHx remaining after degradation and the degradation yield were determined through GC-MS. To prepare the samples for GC-MS, fatty acid methyl ester derivatization was performed. Chloroform and methanol/sulfuric acid (85:15 *v*/*v*) were added at the same volume (1 mL), and methanolysis was conducted for 2 h at 100 °C. Subsequently, the samples were cooled to room temperature, and 1 mL of HPLC-grade water was added to the vials, followed by vortexing for 1 min. Subsequently, the organic-phase layer at the bottom of the vials was transferred to a 1.5 mL e-tube containing anhydrous sodium sulfate (${Na}_{2}{SO}_{4}$) to eliminate residual water. The samples were filtered (0.2 μm pore size) and injected into the GC-MS equipment (YoungIn ChroMass, Anyang, Republic of Korea) using a fused silica capillary column (Elite-5 ms, 30 m × 0.25 mm i.d. × 0.25 μm). Subsequently, the samples were exposed to a linear temperature gradient for analysis for an initial 1 min at 50 °C, followed by a linear increase of 15 °C/min up to 120 °C; at this stage, the temperature was maintained for an additional 2 min. Finally, the temperature was further increased by 10 °C/min up to 300 °C; at this stage, the temperature was maintained for 10 min [65]. The injector-port temperature was maintained at 250 °C. The mass spectra were obtained using electron impact ionization at 70 eV, and the scan spectra were recorded in the range of 45–450 nm. Detection and fragmentation analyses of the major products were performed using selected-ion monitoring. A calibration curve was established to estimate the quantity of residual blend copolymer films.

## 3. Results and Discussion

### 3.1. Determination of 3HHx Mole Fractions of P(3HB-co-3HHx)

*C. necator* H16 was fed with bean oil to provide fatty acids and stimulate the P(3HB) production in a 3 L working volume in a 5 L fermenter for 140 h (Figure S1). Comparatively, Re2668/pCB81 was inoculated to produce P(3HB-*co*-20 mol%3HHx) in a 3 L working volume in a 5 L fermenter (for 132 h). To produce the blended films of P(3HB-*co*-3HHx) with various mole fractions of 3HHx, the amount of each polymer required for blending was determined by considering the molecular number and molecular weight of the monomers in each polymer. The mole fraction of 3HHx in the copolymers was determined through GC, based on which P(3HB) and P(3HB-*co*-20 mol%3HHx) were blended to create P(3HB-*co*-3HHx) with mole fractions of 3HHx ranging from 3.69 to 15.11 mol% (Table 3). The process of blending each film required approximately 2 h, and blending was relatively easy owing to the good miscibility of the materials. The DSC results demonstrated a single $T_{g}$ for the blend, indicating good compatibility between the two polymers (Figure S2). Four blended films prepared by using this method were used in the subsequent experiments.

### 3.2. Comparison of Original and Blended P(3HB-co-3HHx) with FT-IR, NMR and UV Spectra

**Figure 2:**
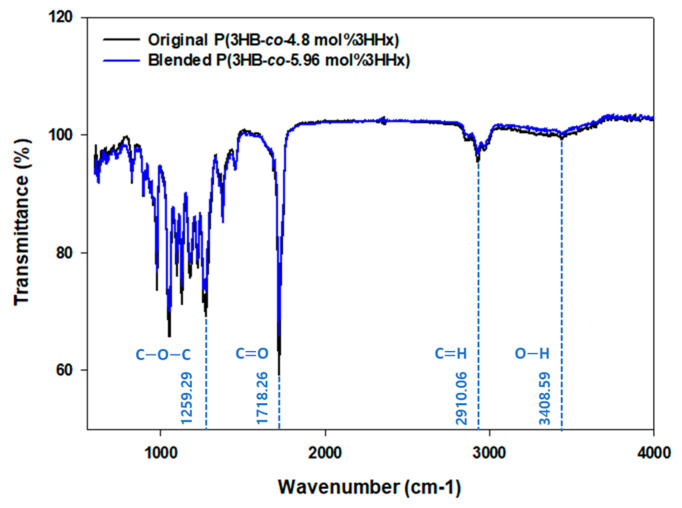
FT-IR spectrum of original P(3HB-*co*-4.8 mol%3HHx) produced by Re2668/pCB81 and blended P(3HB-*co*-5.96 mol%3HHx).

To verify the availability of blended P(3HB-*co*-3HHx), the properties of original P(3HB-*co*-4.8 mol%3HHx) obtained from microorganism alone and blended P(3HB-*co*-5.96 mol%3HHx) were compared. Per FT-IR analysis results, the 1718.26 cm^−1^ peak is the characteristic peak of the ester bond (C=O) [66], the main component of the P(3HB-*co*-3HHx) polymer, which is clearly seen in both samples. This peak was similar in both samples, which confirmed that the original chemical structure was maintained during the blending process. In addition, the 1259.29 cm^−1^ peak is the C–O–C peak, and there was no significant difference in this peak in both the blend and the original polymer, confirming that the two polymers were well mixed [67]. The 2910.06 cm^−1^ peak is mainly related to methylene groups (C–H), which represents the alkane component of the PHA structure, and it appeared similarly in both spectra (Figure 2).

**Figure 3:**
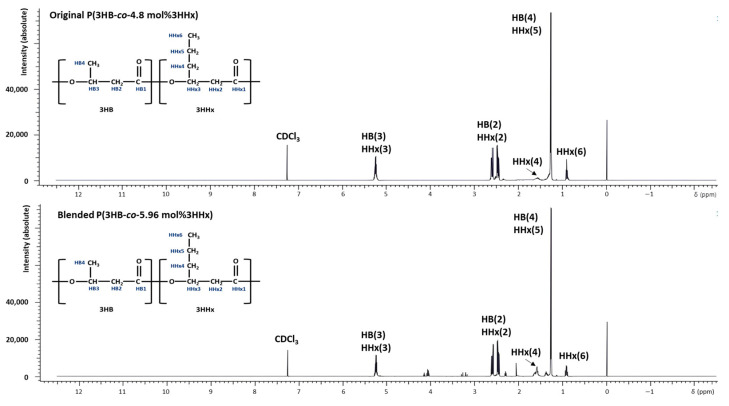
1H-NMR spectrum (500 MHz) of original P(3HB-*co*-4.8 mol%3HHx) produced by Re2668/pCB81 and blended P(3HB-*co*-5.96 mol%3HHx).

Also, we performed and compared original P(3HB-*co*-4.8 mol%3HHx) and blended P(3HB-*co*-5.96 mol%3HHx) using ^1^H NMR analysis and the presence of the 3HB and 3HHx monomers and the composition of the extracted copolymer was confirmed (Figure 3). As a result, both samples were confirmed to have the presence of protons of 3HB and 3HHx monomers at the same ppm in the remaining peaks, except for the CDCl_3_ solvent peak at δ = 7.3 ppm [68]. When we compared the original and blended films using ^13^C NMR analysis peaks were observed at 20 ppm, 41 ppm, 68 ppm, and 170 ppm for both samples, supporting FT-IR and ^1^H-NMR.

Additionally, we compared the UV spectra of original P(3HB-*co*-4.8 mol%3HHx) and blended P(3HB-*co*-5.96 mol%3HHx) using UV-Vis (Figure S3). As a result, both samples were confirmed to absorb near 220–250 nm due to functional groups such as carbonyl groups (–C=O) [69]. The appearance of absorption in a similar wavelength range in these spectra reveals that the two samples have similar structural features.

### 3.3. GPC of P(3HB-co-3HHx) Blends

The molecular weights of P(3HB-*co*-3HHx) copolymers containing various 3HHx monomer fractions were analyzed via GPC (Table 4). The $M_{w}$, $M_{n}$, and Đ values of blended P(3HB-*co*-3HHx) were measured, among which the highest $M_{w}$ was 28.63 × 10^4^, and the relevant copolymer contained 3.69 mol% of the 3HHx monomer. As the 3HHx mole fraction of the blended copolymers increased from 3.69 to 15.11 mol%, their molecular weights decreased from 28.63 × 10^4^ to 24.03 × 10^4^ owing to the low M_w_ of P(3HB-*co*-20 mol%3HHx) produced via microbial fermentation. This is due to the decreased flux caused by the deletion of *phaB*, namely, (R)-3-hydroxybutyryl-CoA dehydrogenase; this affected the flux originating from acetoacetyl-CoA to (R)-3-hydroxybutyryl-CoA, whose deletion decreased the speed of production and the molecular weight of the polymer in *C. necator* [70]. These results are similar to those of previous studies showing that an increase in the 3HHx mole fraction is accompanied by a decrease in $M_{w}$ [71]. Reducing the molecular weight of a polymer can enhance its ductility, resulting in improved elasticity and shock-absorption capability [72]. Consequently, this enhancement may increase the flexibility and biodegradability of the blended polymer and simplify processing [73,74]. However, blended P(3HB-*co*-3HHx) presented a more constant M_w_ than the original P(3HB-*co*-3HHx) with various mole fractions of 3HHx.

The Đ values of the blends were higher than those of P(3HB) or P(3HB-*co*-3HHx) before blending. As the molecular weight and molecular weight distribution of each polymer varied, the polymer chain length and distribution of the mixtures were different. This increased the polymer molecular weight distribution of the mixture, leading to an increase in Đ [75]. In addition, Đ likely increased owing to the slight thermal degradation during the film-formation process, which reduces the molecular weight and forms short chains and oligomers [76]. The complex hydrothermal degradation behavior could be influenced by various factors: the hydrophobicity of the material, the steric hindrance due to the propyl group in the HHx unit, the promotional effects of low crystallinity, and easy steam diffusion into the more flexible amorphous regions of P(3HB-*co*-3HHx), resulting in a significant increase in Đ [77]. Conversely, although Đ increased after blending, this increase was an inevitable phenomenon that occurred during the blending process.

### 3.4. Thermal Characterization of P(3HB-co-3HHx) Blends

DSC was performed to assess the thermal characteristics of the blends. The effect of increasing the mole fractions of 3HHx was evaluated by analyzing the changes in $T_{g}$, $T_{m}$, and crystallization abilities of the blends. The first heating cycle was conducted to erase any thermal history associated with the previous processing of the copolymers, and the second heating cycle was performed to assess any remaining curing behavior from the first cycle. As a result, as the 3HHx mole fraction increased from 3.69 to 15.11 mol% 3HHx, the $T_{g}$ and Δ*H* values decreased and $T_{c}$ increased (Table 5). As the 3HHx mole fraction increased, $T_{g}$ tended to decrease from −2.0 to −9.1 °C. $T_{g}$ is typically defined as the temperature region at which the polymer transforms from a glassy, hard material to one that is soft, flexible, and rubbery [78,79]. The subsequent weakening of the cohesive forces between the adjacent polymer chains promotes molecular rotation, leading to a concurrent reduction in $T_{g}$. Moreover, this trend indicated that increasing the average side-chain length led to a decrease in the $T_{g}$. It is often necessary to reduce the $T_{g}$ of polymers to enhance their flexibility, processability, and overall utility [80]. Increasing the amount of 3HHx monomers in the blended P(3HB-*co*-3HHx) reduced the Δ*H* from 75.3 to 37.5 mJ/mg. A similar pattern was observed for the $T_{g}$ value of the blends, which decreased as the 3HHx mole fraction of 3HHx increased. This tendency indicates that a higher mole fraction of 3HHx results in an enhancement in the amorphous character of the copolymer [81]. Moreover, as the 3HHx mole fraction increased from 3.69 to 15.11 mol%, the $T_{c}$ value tended to increase from 43 to 51.5 °C, reflecting a reduction in the crystallization rate. However, the $T_{m}$ value did not constantly increase with the increase in the mole fraction of 3HHx.

### 3.5. Mechanical Properties of P(3HB-co-3HHx) with Various Fractions of 3HHx Monomer

Mechanical tests were conducted using the UTM to characterize the mechanical properties of the P(3HB-*co*-3HHx) films with increasing 3HHx mole fractions produced by blending (Table 6). As the mole fraction of 3HHx increased, the tensile strength and Young’s modulus decreased. As the mole fraction of 3HHx increased from 3.69 mol% to 15.11 mol%, the tensile strength decreased from 5.85 to 3.62 MPa, and Young’s Modulus also decreased from 60.60 to 18.03 MPa. This decrease was attributed to the reduced molecular weight and crystallinity of the blends. The mechanical test results revealed enhanced elasticity in the blended films with higher mole fractions of 3HHx. The EL of P(3HB-*co*-3.69 mol%3HHx) to P(3HB-*co*-15.11 mol%3HHx) continuously increased from 129.34 to 298.87%. Previous studies have shown that the EL tends to increase as the $T_{g}$ value decreases [82]. As the mole fraction of 3HHx in the blend increased, the elasticity and EL of the blend increased. The EL of blended P(3HB-*co*-3HHx) was higher than that for P(3HB). Blending P(3HB-*co*-20 mol%3HHx) with P(3HB) induced an effect similar to that imparted by the addition of a plasticizer. The augmented entanglement facilitated by the HHx side chain appeared to adequately offset the reduced crystallinity, thereby maintaining a nearly constant tensile strength [83]. As a result, by blending P(3HB) and P(3HB-*co*-20 mol%3HHx), the low EL (%) of P(3HB) can be compensated for preparing blended P(3HB-*co*-3HHx) with significantly high EL values.

### 3.6. Degradation of Blended P(3HB-co-3HHx) by Bacteria Strains

#### 3.6.1. Clear Zone Test to Confirm Biodegradability of Blended P(3HB-*co*-3HHx)

**Figure 4:**
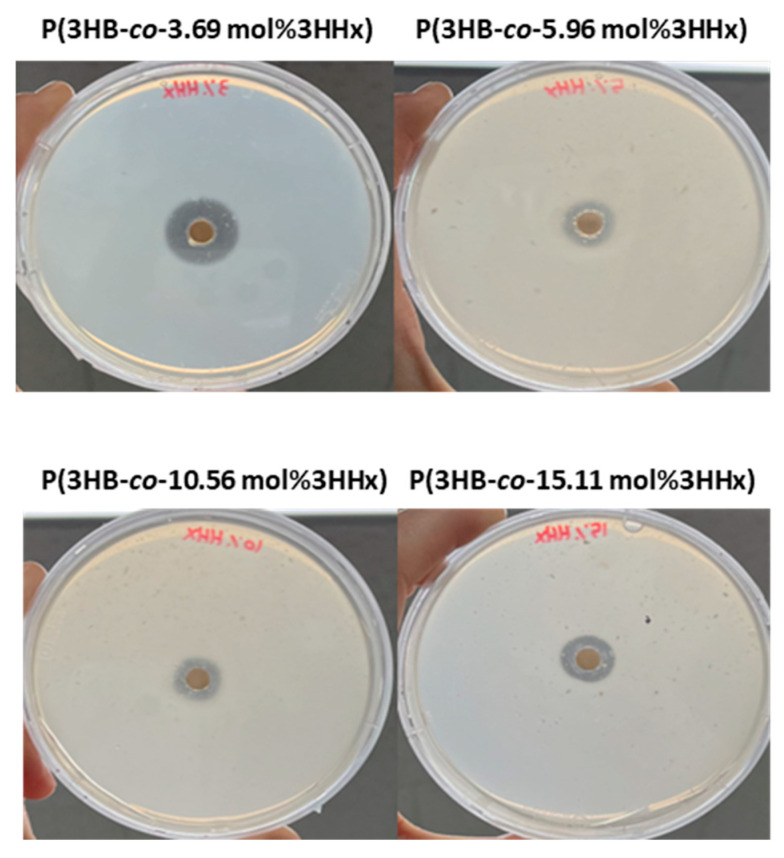
Clear zone test results for the different P(3HB-*co*-3HHx) samples containing various 3HHx contents using *Microbulbifer* sp. SOL66.

To confirm the biodegradability of P(3HB-*co*-3HHx), we selected the *Microbulbifer* sp. SOL66, which has a high degradation ability for PHA polymers. Clear zone tests were conducted to confirm the degradability of the blended polymer, which is a frequently used approach to identify organisms with the ability to degrade a particular polymer [84]. Approximate estimates can be obtained by observing the formation of transparent zones. Solid plates were prepared using P(3HB-*co*-3HHx) emulsions with 3.69–15.11 mol% 3HHx. After inoculating the paper discs above the solid plates with the *Microbulbifer* sp. SOL66, the plates were incubated at 37 °C for 5 days, after which clear transparent zones formed around the paper discs in all four solid plates (Figure 4), confirming the degradation of the prepared blended P(3BH-*co*-3HHx).

#### 3.6.2. Degradation of of P(3HB-*co*-3HHx) with Various 3HHx Mole Fractions

**Figure 5:**
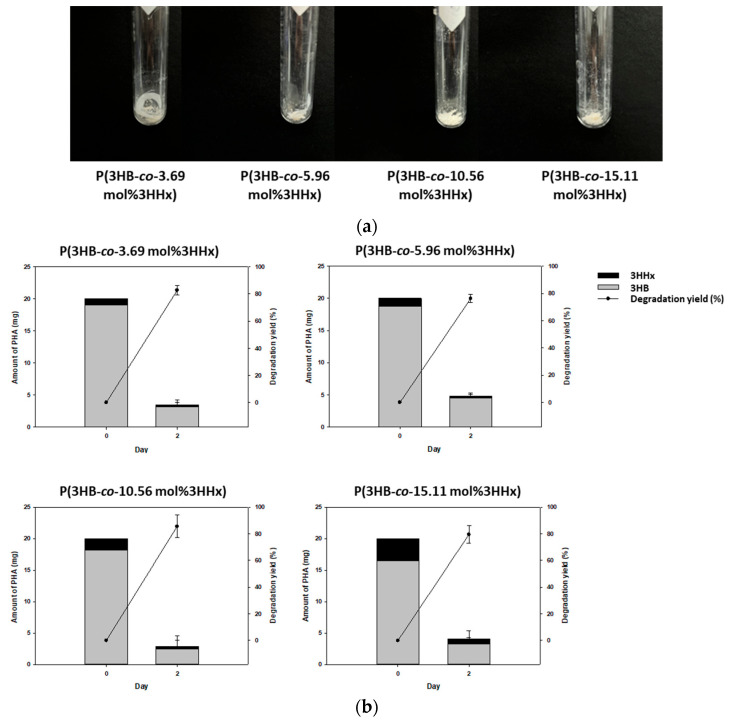
P(3HB-*co*-HHx) film degradation in the liquid medium by *Microbulbifer* sp. SOL66. (**a**) Visual appearance of the blended P(3HB-*co*-3HHx) films undergoing alterations after degradation. (**b**) Gas chromatography–mass spectrometry results for the degradation yields (%) of the blended P(3HB-*co*-3HHx) films with different 3HHx mole fractions.

To determine whether the increase or decrease in the mole fraction of 3HHx affects biodegradability, degradation by *Microbulbifer* sp. SOL66 of blended P(3HB-*co*-3HHx) was tested. During this experiment, 20 mg blended films with various 3HHx mole fractions were added to the MB liquid medium and incubated at 37 °C. The blended films were degraded by *Microbulbifer* sp. SOL66 over a period of 2 days, and morphological alterations were subsequently noted to lyophilize the remaining blended P(3HB-*co*-3HHx) films after degradation (Figure 5a). After 2 days of degradation, all four blended films were degraded, demonstrating a significant reduction in the amount of the blended P(3HB-*co*-3HHx) films.

Subsequently, the degradation yield of the blended films was determined through GC-MS. The results indicated that the degradation yields for P(3HB-*co*-3.69 mol%3HHx), P (3HB-*co*-5.96 mol%3HHx), P (3HB-*co*-10.56 mol%HHx), and P(3HB-*co*-15.11 mol%3HHx) were 82.64%, 76.28%, 85.58%, and 79.53%, respectively (Figure 5b). All films were degraded and showed a degradation yield of >76% over 2 days. The highest degradation yield was achieved by the blended film with a 10.56 mol% mole fraction of 3HHx; however, no particular degradation pattern was observed with the increase in the mole fraction of 3HHx. In addition, the mole fraction of 3HHx had a limited effect on the degradability of blended P(3HB-*co*-3HHx). As the 3HHx mole fraction increases, the crystallinity decreases and the degradation is promoted, but above a certain level, the decrease in crystallinity may no longer have a significant effect on the degradation efficiency [85]. Especially in the fast degradation test like *Microbulbifer* sp. SOL66, the difference in degradation speed was not significant once the enzyme accessibility was secured over a certain level, and the degradation rate may not increase in proportion to the 3HHx mole fraction [86,87].

## 4. Conclusions

The utilization of biodegradable bioplastics, particularly PHAs, is a promising solution to address the growing concerns regarding environmental pollution caused by conventional plastic materials. Our study focused on the production of blended P(3HB-*co*-3HHx) copolymers with different mole fractions of 3HHx achieved by mixing P(3HB) and P(3HB-*co*-20 mol%3HHx) produced by microorganisms and their characterization.

The original P(3HB-*co*-3HHx) without blending was compared with the blended P(3HB-*co*-3HHx), and the FT-IR analysis results showed that the chemical structure did not change significantly after blending, and the NMR analysis confirmed that both samples had the same 3HB and 3HHx monomer protons, confirming that the blending was structurally similar to the original P(3HB-*co*-3HHx). After that, the mechanical and thermal properties of blended P(3HB-*co*-3HHx) with various 3HHx mole fractions were investigated. Blended P(3HB-*co*-3HHx) demonstrated a constant $M_{w}$ value in relation to original P(3HB-*co*-3HHx) with various mole fractions of 3HHx. In addition, the EL increased as the $T_{g}$ value decreased in the mole fraction of 3HHx. The biodegradability of the blended copolymer was further confirmed via a degradation analysis conducted using *Microbulbifer* sp. SOL66, which demonstrated a large degradation yield of >76% in only 2 days and thus highlighted the ecofriendly characteristics of these materials. By adjusting the 3HHx monomer content during the blending of P(3HB) and P(3HB-*co*-3HHx), blended P(3HB-*co*-3HHx) exhibited properties similar to those of original P(3HB-*co*-3HHx) with various 3HHx contents, confirming that production by the blending method was an alternative way of producing P(3HB-*co*-3HHx) with a specific 3HHx fraction. The method of blending P(3HB) and P(3HB-*co*-3HHx) is expected to be useful and produces P(3HB-*co*-3HHx) with more controlled composition and properties.

## Figures and Tables

**Table 1:** P(3HB-*co*-3HHx) production by various strains.

Strains	3HHx (mol%)	PHA Titer (g/L)	Ref.
*Escherichia* *coli*	13.2	12.9	[25]
*Aeromonas caviae*	25	27	[26]
*Aeromonas eutrophus* (PHB⁻4/pJRDEE32d13) harboring *pha* $C_{AC}$	4	76	[27]
*Cupriavidus necator* Re2058/pCB113	19	73	[28]
*Pseudomonas putida* ${GP}_{p104}$ harboring *pha* ${C1}_{Ps}$	16	43	[29]
*Halomonas bluephagenesis* TDC-CJ harboring *pha* $C_{AC}$ and *pha* $J_{AC}$	14.21	55	[30]

**Table 2:** Strains and plasmids used in this study.

Strain or Plasmid	Description	Ref.
Bacterial strains		
*C. necator* H16	Wild-type strain, Gm resistant	[46]
Re2668	Mutant from DSM 530. Chemolithotrophic gromolh with hydrogen. Constitutive G-6-PDH. Purchasing from Korean Collection for Type Cultures (KCTC)	KCTC 2668
*Microbulbifer* sp. SOL66	High polyhydroxyalkanoates (PHA)-degrading strain	[45]
Plasmids		
pCB81	pBBR1MCS-2-based plasmid with *phaC_Ra_* from *Rhodococcus aetherivorans*, *phaJ_pa_* from *Pseudomonas aeruginosa*, and *phaA_Re_* from *Cupriavidus necator*	[47]

**Table 3:** Blending ratios of the blended films.

Blending Ratio of the Blended Film		
Samples	P(3HB) (g)	P(3HB-*co*-3HHx) (g)
P(3HB-*co*-3.69 mol%3HHx)	0.5	0.186
P(3HB-*co*-5.96 mol%3HHx)	0.5	0.352
P(3HB-*co*-10.56 mol%3HHx)	1	0.527
P(3HB-*co*-15.11 mol%3HHx)	1	1.587

**Table 4:** Gel permeation chromatography (GPC) analysis of the P(3HB-*co*-3HHx) copolymer containing various 3HHx mole fractions.

	Samples	$M_{w}\times 10^{4}$	$M_{n}\times 10^{4}$	Đ
Original P(3HB-*co*-3HHx) from microorganisms alone	P(3HB)	84.3	62.6	1.35
	P(3HB-*co*-4.8 mol%3HHx)	49.46	18.43	2.68
	P(3HB-*co*-9.8 mol%3HHx)	32.6	6.50	4.97
	P(3HB-*co*-20 mol%3HHx)	3.40	1.60	2.10
Blended P(3HB-*co*-3HHx)	P(3HB-*co*-3.69 mol%3HHx)	28.63	4.72	6.06
	P(3HB-*co*-5.96 mol%3HHx)	25.12	7.87	4.21
	P(3HB-*co*-10.56 mol%3HHx)	24.82	4.97	5.84
	P(3HB-*co*-15.11 mol%3HHx)	24.03	7.08	4.67

**Table 5:** Characteristics of the P(3HB-*co*-3HHx) blended films determined via differential scanning calorimetry.

Samples	$T_{g}$ (°C)	$T_{m}$ (°C)	Δ*H* (mJ/mg)	$T_{c}$ (°C)
Original P(3HB)	7.5	171.0	83.9	68.8
Blended P(3HB-*co*-3.69 mol%3HHx)	−2.0	169.3	75.3	43.0
Blended P(3HB-*co*-5.96 mol%3HHx)	−7.5	161.4	68.6	43.8
Blended P(3HB-*co*-10.56 mol%3HHx)	−8.1	169.4	40.8	52.9
Blended P(3HB-*co*-15.11 mol%3HHx)	−9.1	172.1	37.5	51.5
Original P(3HB-*co*-20 mol%3HHx)	−9.3	171.0	27.3	62.9

$T_{g}$: glass transition temperature, $T_{m}$: melting temperature, Δ*H*: enthalpy change of melting temperature, $T_{c}$: crystallization temperature.

**Table 6:** Mechanical test results for blended P(3HB-*co*-3HHx) with increasing HHx mole fraction.

Samples	Tensile Strength [MPa]	Young’s Modulus [MPa]	EL [%]
Original P(3HB)	24.83	545.39	9.93
Blended P(3HB-*co*-3.69 mol%3HHx)	5.85	60.60	129.34
Blended P(3HB-*co*-5.96 mol%3HHx)	5.39	55.57	122.41
Blended P(3HB-*co*-10.56 mol%3HHx)	4.49	23.18	267.89
Blended P(3HB-*co*-15.11 mol%3HHx)	3.62	18.03	298.87
Original P(3HB-*co*-20 mol%3HHx)	1.82	10.77	197.14

## Data Availability

The original contributions presented in this study are included in the article/Supplementary Material. Further inquiries can be directed to the corresponding authors.

## Supplementary Materials

The following supporting information can be downloaded at: https://www.mdpi.com/article/10.3390/polym16213077/s1, Figure S1: 3 L scale fermentation to produce the homopolymer P(3HB). (a) optical density (595 nm) according to fermentation time. (b) DCW and PHA content according to fermentation time; Figure S2: DSC analysis for blended P(3HB-*co*-3HHx) containing various 3HHx contents; Figure S3: UV-Vis spectra analysis. (a) Original P(3HB-*co*-4.8 mol%3HHx) and (b) blended P(3HB-*co*-5.96 mol%3HHx).
