# Supplementary material for: Evaluation of Blended Poly(3-hydroxybutyrate-co-3-hydroxyhexanoate) Properties Containing Various 3HHx Monomers
Source: Polymers (Basel). 2024 Oct 31;16(21):3077. doi: 10.3390/polym16213077 (PMC11548210; doi:10.3390/polym16213077)
Supplement: Supplementary file 1 [file polymers-16-03077-s001.zip › polymers-3261328-supplementary.pdf]

Evaluation of blended Poly(3-hydroxybutyrate-*co*-3-hydroxyhexanoate) properties containing various 3HHx monomers

Supplementary Information

Supplementary Figure S1

(a)

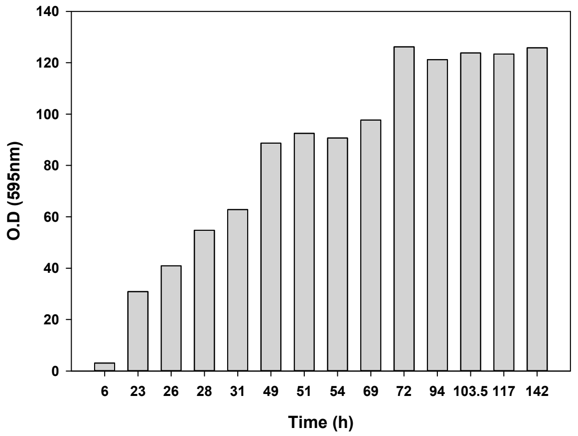

(b)

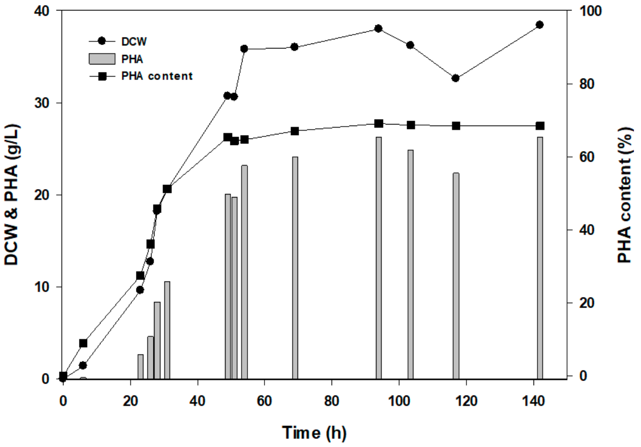

Figure S1. 3 L scale fermentation to produce the homopolymer P(3HB). (a) optical density (595 nm) according to fermentation time. (b) DCW and PHA content according to fermentation time.

Supplementary Figure S2

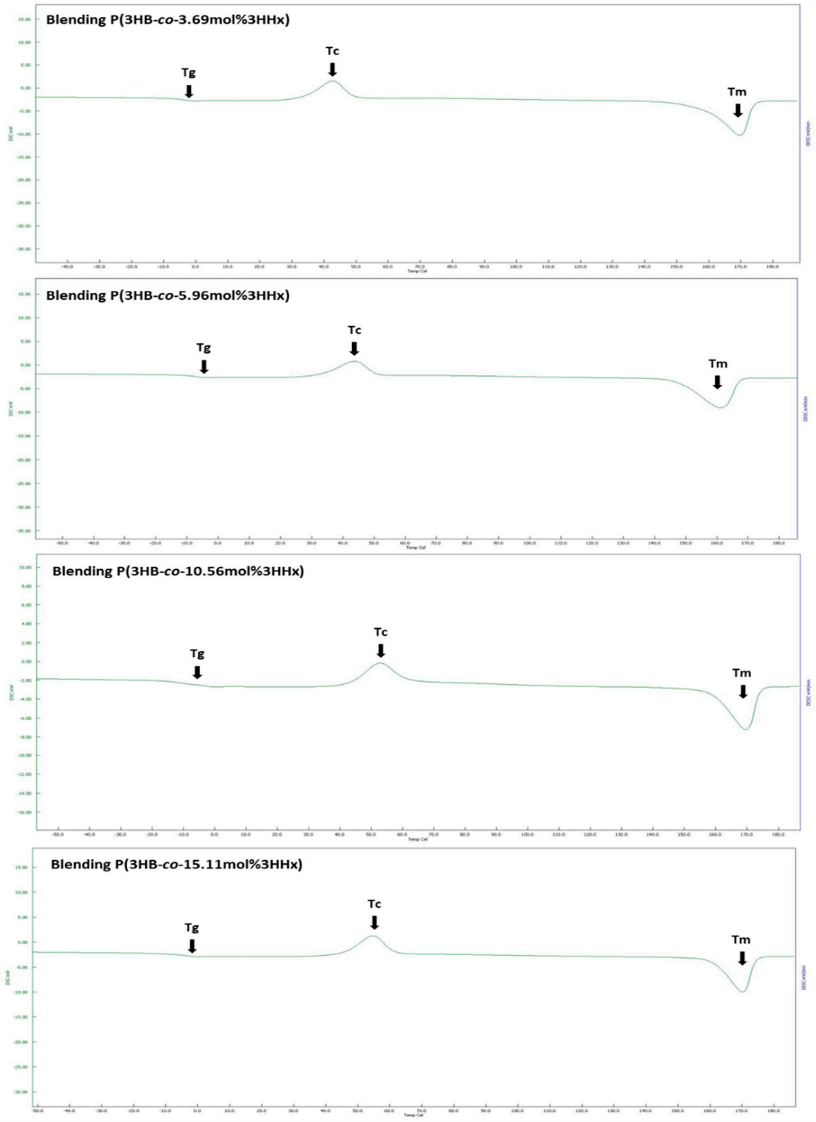

Figure S2. DSC analysis for blended P(3HB-*co*-3HHx) containing various 3HHx contents.

Supplementary Figure S3

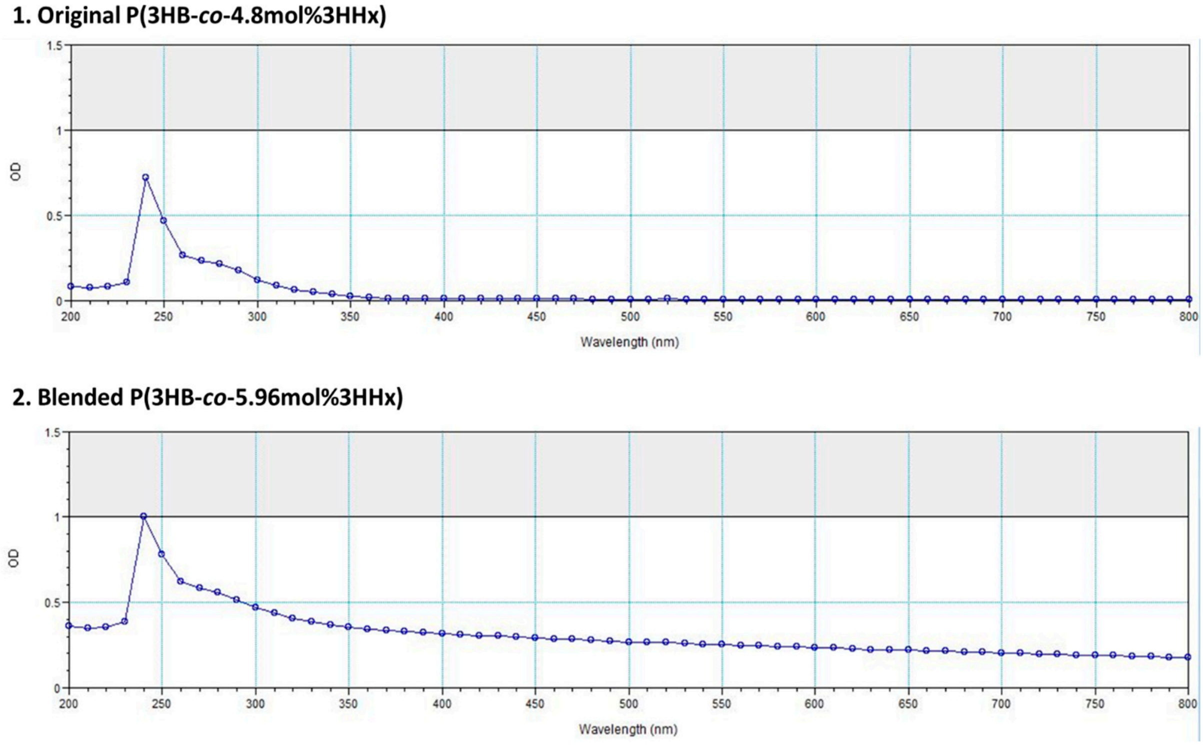

Figure S3. UV-Vis spectra analysis. Original P(3HB-*co*-4.8 mol%3HHx) and blended P(3HB-*co*-5.96 mol%3HHx).
